# Humoral and Cellular Responses to COVID-19 Vaccination Indicate the Need for Post-Vaccination Testing in Frail Population

**DOI:** 10.3390/vaccines10020260

**Published:** 2022-02-08

**Authors:** Wojciech Witkowski, Sarah Gerlo, Evelien De Smet, Magdalena Wejda, Delphine Acar, Steven Callens, Stefan Heytens, Elizaveta Padalko, Hanne Vercruysse, Piet Cools, Linos Vandekerckhove

**Affiliations:** 1 HIV Cure Research Center, Department of Internal Medicine and Pediatrics, Ghent University Hospital, Ghent University, 9000 Ghent, Belgium; wojciech.witkowski@ugent.be (W.W.); sarah.gerlo@ugent.be (S.G.); Evelien.DeSmet@uzgent.be (E.D.S.); magdalena.wejda@ugent.be (M.W.); delphine.acar@ugent.be (D.A.); 2 Department of Biomolecular Medicine, Faculty of Medicine and Health Sciences, Ghent University, 9000 Ghent, Belgium; 3 Department of Internal Medicine and Pediatrics, Ghent University Hospital, Ghent University, 9000 Ghent, Belgium; Steven.Callens@UGent.be; 4 Department of Public Health and Primary Care, Faculty of Medicine and Health Sciences, Ghent University, 9000 Ghent, Belgium; stefan.heytens@ugent.be; 5 Department of Diagnostic Sciences, Faculty of Medicine and Health Sciences, Ghent University, 9000 Ghent, Belgium; elizaveta.padalko@UGent.be (E.P.); Piet.Cools@UGent.be (P.C.); 6 Department of Medical Microbiology, Ghent University Hospital, 9000 Ghent, Belgium; 7 Research and Analytics, Liantis Occupational Health Services, 8000 Bruges, Belgium; hanne.vercruysse@liantis.be

**Keywords:** SARS-CoV-2, nursing home, vaccination, antibodies, cellular immunity

## Abstract

Despite the high efficacy of the BNT162b2 vaccine in the general population, data on its immunogenicity among frail elderly individuals are limited. Recently, levels of anti-SARS-CoV-2 spike IgG antibodies and serum neutralization titers were confirmed as good immune markers of protection against the virus, with evidence showing a reverse correlation between these two parameters and susceptibility to infection. Here we analyzed sera from 138 nursing home residents (median age of 88.9 years) and 312 nursing home staff (median age of 50.7 years) to determine the humoral response to two doses of the BNT162b2 vaccine, and found markedly decreased serum anti-spike antibody levels and neutralization titers in the nursing home resident (NHR) group, with over 11% non-responders compared to only 1.3% among the controls. Moreover, three months post-vaccination, a significant decrease in antibody titers was observed in COVID-19-naive nursing home residents. Subsequent flow cytometry and interferon gamma secretion analyses indicated that antibody non-responders among NHRs also failed to mount cellular responses. The presented data emphasize that additional measures are needed in the population of frail elderly individuals. Given the high proportion of non-responders among NHRs, continued monitoring should be considered in this group.

## 1. Introduction

The COVID-19 pandemic has disproportionately affected the elderly and frail populations, who accounted for the majority of deaths [1]. The unprecedented speed of the development and enrollment of the emergency-authorized vaccines [2,3,4], together with the immediately observed real-life effectiveness in preventing new infections [5,6], provided much-needed hope that the social restraints imposed all around the world could soon be lifted. However, the question remained as to how the most vulnerable groups, which for obvious reasons could not be enrolled in Phase I–III clinical trials, would respond to the vaccination. Real-world data demonstrating post-vaccination antibody responses in the groups most susceptible to SARS-CoV-2-related morbidity and mortality remain limited. Observations from the few available studies in nursing home residents show conflicting results, with some reporting very good immunogenicity following the administration of two doses [7,8] and others indicating decreased levels of seroconversion and neutralization [9,10]. Anti-spike IgG antibody levels and their neutralization potential have been correlated with vaccine efficacy in preventing breakthrough infections [11], and in the context of variants of concern, like the B.1.617.2 strain designated as Delta [12] and—to a much lesser degree—Omicron [13]. Early reports on vaccine efficacy in the most vulnerable groups indicated that breakthrough infections occur in a small percentage of vaccine recipients, with nursing home residents becoming disproportionally more affected than the staff [14]. The fatality ratio of SARS-CoV-2 infection is directly correlated with age, and is highest in the elderly population. In Belgium, most COVID-19-related deaths occurred in NHRs, and consequently this group was prioritized in the immunization campaign that started shortly after the market authorization of the Comirnaty BNT162b2 vaccine (Pfizer-BioNTech, Mainz, Germany) in Europe. By the end of March 2021, close to 100% of Belgian NHRs had received a second dose of the BNT162b2 vaccine. Given the vulnerability of this group, we decided to conduct a study comparing the post-vaccination antibody response in NHRs to the working-age population of NHS two weeks post-vaccination. Three months later, samples were obtained from a subset of the NHRs and additional assays were performed to assess the cellular response against SARS-CoV-2.

## 2. Materials and Methods

### 2.1. Study Design and Population

Residents (*n* = 138) and staff (*n* = 312) from six nursing homes in Flanders (Belgium) were included in the study from 18 January until 4 March 2021 (baseline visit). Venous blood for serology was obtained at the day of the first dose of the Pfizer-BioNTech vaccination, 14 days later (at the time of second dose administration) and 14 days after the second dose (follow-up visit). At baseline, the presence of previous SARS-CoV-2 infection was determined by assessing anti-NCP and anti-S1 antibodies. At follow-up, anti-S1 and pseudovirus neutralization were assessed. A subset of residents was sampled once more, 3 months after receiving the second vaccination dose. Due to the limited access to the frail population given additional safety measures imposed, we were able to obtain only a limited number of 20 samples for this subset analysis. Collected venous blood was analyzed for the presence of anti-NCP and anti-S1 IgG, pseudovirus neutralization and PBMC response to COVID-19 S peptide stimulation. Sampling timeline overview is presented in Figure S1.

### 2.2. Sample Collection

Serum tubes containing approximately 5 mL of venous blood were obtained from each participant and transported to the Laboratory of Clinical Microbiology of the Ghent University Hospital (Ghent, Belgium) within six hours after sample collection. Upon arrival, serum tubes were centrifuged at 2000× *g* for 8 min and stored at 4 °C. The following day, serum was aliquoted into new serum vials and frozen at −20 °C until further analysis. For the subset of samples obtained 3 months post-vaccination, two K3 EDTA tubes with approximately 10 mL of venous blood were collected from the participants and transported within 4 h to the HIV Cure Research Center at the Ghent University Hospital (Ghent, Belgium). Samples were immediately processed upon arrival, that is, plasma was stored at −20 °C and PBMCs were isolated immediately for use in downstream assays.

### 2.3. Antibody Detection

The presence of SARS-CoV-2 S1 and NCP-specific IgG antibodies was determined using a semi-quantitative ELISA (EUROIMMUN, Lübeck, Germany) according to the manufacturer’s instructions. Results were expressed in optical density (OD) ratio. Throughout the analyses, we considered samples falling within the grey zones of both tests (OD ratio 0.8–1.1) positive.

### 2.4. Pseudovirus Neutralization Assay

Pseudovirions were generated by transiently transfecting HEK293T cells with non-infectious human immunodeficiency virus 1 SG3ΔEnv (NIH HIV Reagent Program, molecular clone cat. no. ARP-11051) and codon optimized, SARS-CoV-2 wild-type S envelope plasmid, generated from pBOB-CAG-SARS-CoV2-Spike-HA vector (a gift from Gerald Pao; RRID: Addgene_141347) by restriction digest with NdeI enzyme (New England BioLabs, Ipswich, UK). Forty-eight hours post-transfection, viral supernatants were collected and 10-fold concentrated by centrifugation in Amicon Ultra-15 MWCO 100 kDa centrifugal filters. Aliquots were stored at −80 °C until further use. TZM-bl cells (NIH HIV Reagent Program, cat. no. ARP-8129) were modified to overexpress ACE2 receptor by lentiviral transduction. Briefly, human ACE2 was subcloned from pDUO2-hACE2-TMPRSS2a vector (InvivoGen) into pTRIP-EF1a-IRES-dNGFR lentiviral vector backbone. ACE2-expressing cells were bulk-sorted following staining with His-tagged, recombinant SARS-CoV-2 receptor-binding domain (a kind gift from Prof. Xavier Saelens) and anti-His-PE (Miltenyi Biotec.).

To measure the extent of neutralization, TZM-bl/ACE2 cells were infected with pseudovirus generated as described above in the presence of heat-deactivated (56 °C, 30 min) human serum. For each sample, 6 consecutive, 3-fold dilutions in phosphate-buffered saline (PBS) were prepared. Briefly, TZM-bl/ACE2 cells at 10,000 cells/well were added to serially diluted sera after incubation with pseudovirus at 37 °C for 30 min in a 96-well plate in the presence of Polybrene (Merck Millipore) at a final concentration of 5 µg/mL and spin-inoculated at 32 °C and 2300 rpm for 30 min. The cell culture medium was refreshed after 18 h. Two days post-infection, cells were washed once with PBS and incubated for 30 min at 37 °C with colorimetric β-Galactosidase Assay Reagent (Thermo Scientific, Rockford, IL, USA). Absorbance at 405 nm was measured using a Multiskan FC microplate spectrophotometer (Thermo Scientific). The highest serum dilution resulting in 50% pseudovirus neutralization was determined by non-linear regression curve fitting using GraphPad Prism version 9.1.

### 2.5. T-Cell Response to SARS-CoV-2 Spike Peptide Pool

PBMCs were isolated from EDTA blood by density gradient centrifugation and immediately plated in a 96-well U-bottom plate at 1 million cells/well in 100 µL of RPMI medium (Gibco) containing 5% human pooled AB serum (Cellect) and penicillin/streptomycin (Gibco) in cell culture incubator (37 °C, 5% CO_2_). After 2 h, cells were stimulated with a pool of spike peptides at 6 nmol each, spanning the whole protein sequence (GenBank MN908947.3, Protein QHD43416.1) and obtained from Miltenyi Biotec (Bergisch Gladbach, Germany). For each donor a negative DMSO control and a positive stimulation control (Cytostim, Mitenyi Biotec) were also used in separate wells. After 16 h, Brefeldin A (Miltenyi Biotec) was added, and incubation continued for 4 additional hours after which the cell culture supernatant was collected and frozen at −80 °C and the cells were resuspended in PBS. Subsequently, a live/dead staining was performed with 405/452 Fixable Dye (Miltenyi Biotec) followed by fixation and permeabilization with Inside Fix and Inside Perm solutions according to manufacturer’s guidelines (Milenyi Biotec). CD3-APC Antibody (Clone REA613), CD4-Vio Bright 515 (Clone REA623), CD8-VioGreen (Clone REA734), CD69-PE (Clone REA824), TNFα-PE Vio 770 (Clone REA656) and CD154-APC Vio 770 (Clone REA238), all purchased from Miltenyi Biotec, were used for staining. Cells were analyzed on MACSquant 10 analyzer (Miltenyi Biotec). IFNγ secreted into the cell culture supernatant was quantified by ELISA (QuantiFERON) according to the manufacturer’s guidelines (Qiagen, Germantown, MD, USA).

### 2.6. Statistical Analysis

**Figure 1:**
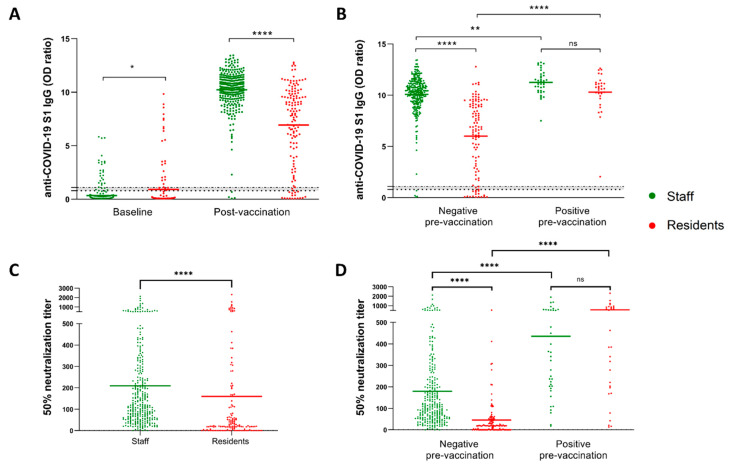
Nursing home residents showed impaired antibody response to two doses of BNT162b2 vaccine. (**A**) Anti-S1 IgG levels at week 2 post-vaccination with the second dose in NHS (*n* = 312) and NHRs (*n* = 138). (**B**) Data as in (**A**) shown in respect to the pre-vaccination anti-SARS-CoV-2 serostatus. (**C**,**D**) Ability of serum collected from NHS and NHRs two weeks post-vaccination to neutralize SARS-CoV-2 pseudovirus. * *p*-value = 0.049, ** *p*-value = 0.0014, **** *p*-value < 0.0001, ns-not significant. Dotted horizontal lines show manufacturer’s determined cutoff level and grey-zone values.

Data was analyzed with GraphPad Prism 9 software. Non-parametric Mann–Whitney U test was used to compare between-group continuous values. Comparisons between multiple continuous values calculated for data sets presented in Figure 1B,D were performed using Kruskal–Wallis test with Dunn’s method to correct for multiple comparisons. We considered statistical significance as a two-tailed *p*-value < 0.05. Confidence intervals were calculated with one sample *t*-test with standard error.

## 3. Results

### 3.1. Nursing Home Residents Showed Impaired Antibody Response and Neutralization Following BNT162b2 Vaccination

We first compared levels of anti-SARS-CoV-2 S1 IgG antibodies between NHS and NHRs. As shown in Figure 1A, a total of 98.7% (95% confidence interval (CI) 97.5% to 99.9%) of NHS seroconverted two weeks after the second BNT162b2 dose, whereas 88.4% (95% CI 83.1% to 93.7%) of NHRs seroconverted (*p* < 0.0001). BNT162b2 vaccination also induced significantly lower levels of antibody response in NHRs (mean OD ratio 6.94, 95% CI 6.32 to 7.56) compared to NHS (mean OD ratio 10.22, 95% CI 10.01 to 10.43) (*p* < 0.0001). In both NHRs and NHS, SARS-CoV-2 infection prior to vaccination verified by the presence of anti-spike (S) and/or anti-nucleocapsid (NCP) IgG class antibodies at baseline (summarized in Figure S2) resulted in significantly higher responses (*p* = 0.0014 for NHS and *p* < 0.0001 for NHRs) (Figure 1B). Next, we analyzed the ability of post-vaccination sera of study participants to neutralize the SARS-CoV-2 pseudovirus displaying the original wild-type S protein. Again, the NHS showed a superior response to vaccination, with approximately 25% better pseudovirus neutralization (*p* < 0.0001). This was calculated by comparing the dilutions of sera still able to neutralize 50% of pseudovirus between the groups. Meanwhile, on average, 209-times diluted sera of NHS still displayed 50% neutralization (95% CI 178.9 to 239.0); for the NHRs the value was equal to 159.8 (95% CI 104.0 to 215.6). The difference was even more prominent when only the previously non-infected individuals were compared. Here, the NHS displayed an average 50% neutralization score (serum dilution) of 179.4 (95% CI 151.9 to 207.0), whereas the NHR score averaged 48.9 (95% CI 31.8 to 66.1) (Figure 1D). Unlike for NHS we observed no differences in pseudovirus neutralization between male and female residents. As expected, age was clearly correlated with the ability to mount effective neutralizing antibody responses among non-infected study participants (Supplementary Figure S3). When we plotted the pseudovirus neutralization data against the results of semi-quantitative anti-S1 IgG ELISA used in the study, a clear correlation was observed (Supplementary Figure S4). Importantly, there was no statistically significant difference in pseudovirus neutralization between staff and residents infected prior to the vaccination (Figure 1D), despite the observed differences in pre-vaccination anti-S1 IgG antibody range between the groups (Supplementary Figure S2).

### 3.2. Pre-Vaccination Infection Status Secured High Antibody Levels 3 Months Post-Vaccination in NHRs

**Figure 2:**
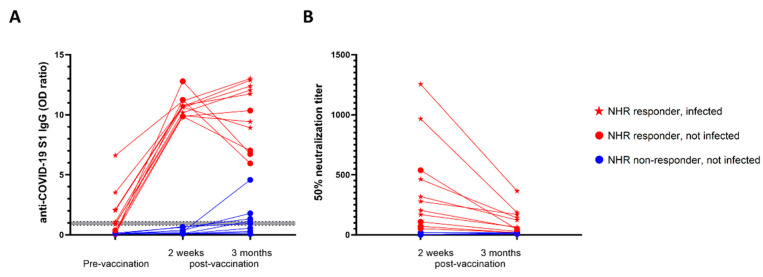
Infection status predicted antibody levels 3 months post-vaccination. (**A**) Evolution of anti-S1 antibody levels in NHRs (*n* = 20) measured at the moment of first vaccine dose administration, and at 2 weeks and 3 months after the second dose. (**B**) Pseudovirus neutralization measured two weeks after the first vaccine dose administration and 3 months after the second dose.

**Figure 3:**
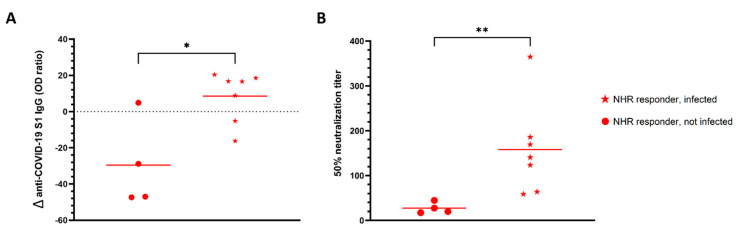
SARS-CoV-2 pre-infection prevented anti-S1 IgG decline. (**A**) Percentage change in anti-S1 IgG levels from two weeks to 3 months after administration of the second dose of vaccine in infected (*n* = 7) and non-infected (*n* = 4) NHR responders. (**B**) Ability of NHR serum to neutralize pseudovirus three months after the second dose of vaccine administration. * *p*-value = 0.02, ** *p*-value = 0.006.

Three months after administration of the second dose, additional blood samples from a subset (*n* = 20) of NHRs were obtained. Nine non-responders, defined as having anti-S1 IgG levels below the cutoff value, and 11 responders were included; among the latter, 7 were infected and 4 were non-infected prior to vaccination. None of the non-responders had been previously infected. As shown in Figure 2 all responders remained positive for anti-S1 IgG at 3 months post-vaccination. Nonetheless, a distinct pattern of antibody level evolution could be seen between non-infected and infected responders. Whereas antibody levels dropped for 3 out of 4 non-infected responders between 2 weeks and 3 months post-vaccination, they modestly increased in 5 out of 7 individuals previously exposed to the virus. Importantly, for all NHRs tested at 3 months post-vaccination, a decrease in neutralizing antibody levels was observed. Despite the small sample size, we found a significant difference for the evolution of anti-S1 IgG levels between non-infected and infected vaccinees, with a trend towards decrease and increase over time, respectively (Figure 3). Moreover, 3 months post-vaccination, pseudovirus neutralization titers were significantly higher in previously infected donors (Figure 3B). Interestingly, five of the nine non-responders at week two post-vaccination became positive at 3 months, but their virus neutralizing capacity remained low (Figure 2B).

### 3.3. Lack of COVID-19-Specific T-Cell Responses among NHR Antibody Non-Responders

**Figure 4:**
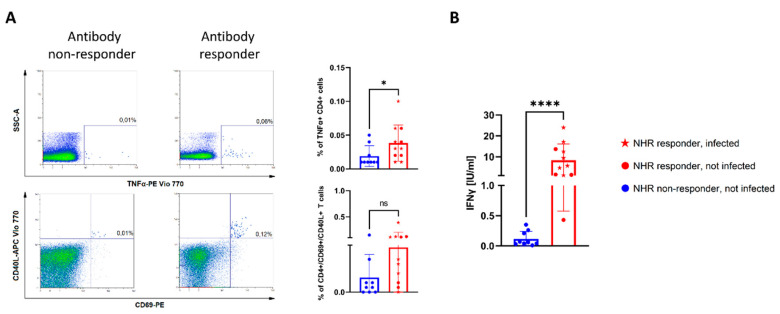
There were no SARS-CoV-2-specific cellular responses in NHRs lacking antibody response to vaccination. (**A**) Dot plots demonstrate typical CD4+ T-cell response to stimulation with a pool of spike peptides in NHR non-responders (left column) and responders (right column) as determined by anti-S1 IgG ELISA. Graphs indicate differences in the percentage of TNFα+ or CD69+/CD40L+, CD4+ T cells. (**B**) Graph indicates differences in IFNγ secretion from NHR (*n* = 20) PBMCs following 22 h stimulation with SARS-CoV-2 spike peptide pool. * *p*-value = 0.04, **** *p*-value < 0.0001, ns-not significant.

Finally, we sought to determine the degree of COVID-19-specific cellular immunity in NHR BNT162b2 recipients. The peripheral blood mononuclear cell (PBMC) response to a COVID-19 spike peptide pool stimulation was tested on the same sub-group of NHRs as described for the 3-month antibody follow-up. Following stimulation, the cells were subjected to flow cytometry analysis with the gating strategy shown in Supplementary Figure S5. Compared to the NHRs who did not mount an anti-S1 IgG antibody response two weeks post-vaccination, antibody responders showed a significantly higher percentage of TNFα-positive, CD4+ T cells (Figure 4A). When cell culture supernatants of peptide-stimulated cells were analyzed for secreted interferon gamma (IFNγ), a clear difference could also be observed, with 0.12 IU/mL (95% CI 0.02 to 0.21) for antibody non-responders and 8.31 IU/mL (95% CI 3.12 to 13.52) for responders (*p* < 0.0001). Three months post-vaccination, levels of secreted IFNγ correlated strongly with pseudovirus neutralization and anti-S IgG levels measured at the same time point (Supplementary Figure S6).

## 4. Discussion

In this study, an in-depth analysis of the antibody response to BNT162b2 vaccination was performed on a sizeable group of 138 nursing home residents that benefited from a control group of 312 staff members who took care of them. For each participant, baseline levels of serum anti-S1 and anti-NCP antibodies were determined to identify previously infected individuals. In accordance with previous reports, past COVID-19 infection resulted in markedly higher anti-S1 antibody following vaccination [15,16], including in NHRs [10,17,18]. Notably, NHRs presented a relatively broad range of post-vaccination antibody titers, while the spread was smaller in the control group. Compared to NHS, residents had on average 32% lower serum antibody levels, with over 10% of NHRs not responding to the vaccination as determined by anti-S1 IgG antibody titers. That stands in contrast with findings from a study of similar size conducted in disabled and frail nursing home residents where only 1 among 134 residents did not show evaluable antibodies [8]. This difference might be explained by the semi-quantitative nature of the test used in our study, where values below a determined cut-off value were considered negative. It could also be explained by the relatively higher average age of our cohort, 88.7 years versus the 82.9 reported from the above-mentioned study.

Importantly, the impaired response to vaccination in NHRs was further reflected in their greatly reduced ability to neutralize SARS-CoV-2 pseudovirus. Like others, we found a correlation between neutralizing antibody (nAb) titers and anti-S IgG antibody titers [11,19], indicating that anti-spike non-responders are not protected by neutralizing antibodies. As expected, pre-vaccination infection status predicted higher titers of neutralizing antibodies, with all of the COVID-19 pre-infected study participants in either group displaying robust pseudovirus neutralization. However, while the COVID-19-naïve staff showed nAb titers approximately 2.5 times lower than their infected counterparts, an over 10-fold difference was observed in the NHR group, in which multiple individuals showed little to no neutralization. The mean nAb titer among the non-infected NHRs was six times lower than that found in NHS. This highlights an extremely different response to vaccination between healthy and frail populations. A recent analysis of the neutralizing antibody response in elderly participants and younger healthcare workers [20] observed that while people over 80 years of age showed reduced response to vaccination, neutralizing antibodies were detected across all age groups after the second dose. Another study showed a more pronounced difference in neutralization when groups of people below 60 and over 80 years of age were compared [21]. In light of a recent report showing that low pseudovirus neutralization titers after vaccination increase the likelihood of breakthrough infections [22], our data clearly indicate that a significant proportion of nursing home residents do not benefit from good levels of protection. Although we did not find statistically significant differences between our study groups when individuals with pre-vaccination COVID-19 were considered, the mean nAb titers were higher in NHRs. Pre-vaccination anti-S1 and anti-NCP IgG levels were lower among the NHS. A limitation of this type of study is that high mortality among the NHRs during the first two waves of COVID-19 pandemic likely resulted in a sampling bias, with only the survivors represented within the group [23]. We also observed a difference in antibody dynamics as a function of pre-vaccination COVID-19 status in a subset of NHRs analyzed 3 months after the second dose administration. The most apparent was a steady level of anti-S1 IgG in most infected subjects, with a marked decline in the COVID-19-naïve participants. Despite the small sample size, the observed differences were statistically significant. Moreover, COVID-19 infection pre-vaccination corresponded with significantly higher nAb titer at 3 months post-vaccination. Notably, several of the non-responders at week 2 reached the cutoff for anti-S1 positivity over the 3-month period, but their neutralization capacity remained undetectable. Additional analysis of T-cell responses performed in the subset of NHRs that had a 3-month follow-up revealed that COVID-19 spike peptide stimulation led to specific activation and secretion of IFNγ, and that this response was more potent in humoral responders and impaired in humoral non-responders.

Data obtained from the T-cell analysis 3 months post-vaccination allows us to make a cautious assumption that upon re-infection, NHRs can mount a T-cell response to the viral antigens, but also suggests that antibody non-responders might not be rescued by T-cell immunity, which was also less potent in these individuals. Evidence from a study comparing the effectiveness of existing vaccines against the B.1.617.2 indicates that BNT162b2 (as well as ChAdOx1 nCoV-19) provide good protection from infection in recipients of both recommended doses [12]. On the contrary, our findings in NHRs indicate that the frail, elderly population does not benefit from the same levels of immune response to vaccination (and consequently protection) as healthy, younger people. A recent report indicating improved reduction of infection and severe illness in the youngest recipients of a booster dose supports that observation [24]. The presented data strongly suggest that routine monitoring of post-vaccination antibody levels is needed to identify over-represented non-responders and antibody waning among NHRs.

## 5. Conclusions

The presented data underscore lower immunogenicity of the BNT162b2 vaccine in SARS-CoV-2 naïve nursing home residents with a clear reduction in the percentage of responders compared to the control group. Vaccinees who did not mount detectable anti-spike antibody also failed to neutralize pseudovirus and showed impaired COVID-19-specific T cell responses.

## 6. Limitations of the Study

Although SARS-CoV-2 infection was monitored by RT-PCR on a weekly basis in our study population, because our assignment to the “negative pre-vaccination” group was based on pre-vaccination IgG measurements, we cannot exclude that we missed some individuals with early infection.

## Data Availability

The data presented in this study are available on request from the corresponding author.

## Supplementary Materials

The following are available online at https://www.mdpi.com/article/10.3390/vaccines10020260/s1, Figure S1: Graphical representation of the study scheme, Figure S2: COVID-19 serostatus before vaccination, Figure S3: Impact of age and sex on pseudovirus neutralization, Figure S4: Correlation between pseudovirus neutralization titers and anti-S1 IgG levels, Figure S5: Gating strategy used for flow cytometry data analysis, Figure S6: Correlation between SARS-CoV-2-specific IFNγ secretion from PBMCs, anti-S1 IgG levels and pseudovirus neutralization.
