# Supplementary material for: Humoral and Cellular Responses to COVID-19 Vaccination Indicate the Need for Post-Vaccination Testing in Frail Population
Source: Vaccines (Basel). 2022 Feb 8;10(2):260. doi: 10.3390/vaccines10020260 (PMC8875521; doi:10.3390/vaccines10020260)
Supplement: Supplementary file 1 [file vaccines-10-00260-s001.zip › vaccines-1540643-supplementary.pdf]

Supplementary Figure S1. Graphical representation of the study scheme. Nursing home staff and residents underwent the same vaccination protocol where the baseline blood sample was collected at the moment of first dose administration followed by second injection two weeks later. After another two weeks blood sample was obtained. For a small subset of residents (*n*=20) another blood draw was performed three months post second dose administration. Created with BioRender.com.

Supplementary Figure S2. COVID-19 serostatus before vaccination. **A)** Pre-vaccination infection status and response to vaccination determined by quantification of anti-S1 IgG and/or anti-NCP IgG levels and applying manufacturer-recommended cut-off values. Percentages refer to the total number of positives among the NHS or NHR groups. **B)** Pre-vaccination anti-S1 IgG and anti-NCP IgG levels among the infected NHS and NHR. *- P value = 0,034. **C-D)** Relation between pre-vaccination anti-S1 IgG and anti-NCP IgG in individual study participants.

Supplementary Figure S3. Impact of age and sex on pseudovirus neutralization. **A)** Mean pseudovirus neutralization titers in serum from NHS or NHR collected 2 weeks post administration of the second vaccination dose. **B)** Graph represents correlation between age and pseudovirus neutralization titers in serum of non-infected NHS or NHR. . * - P value = 0.041, *** - P value = 0.0006, **** - P value <0.0001.

Supplementary Figure S4. Correlation between pseudovirus neutralization titers and anti-S1 IgG levels measured in serum 2 weeks after administration of second dose of the vaccine.

Supplementary Figure S5. Gating strategy used for flow cytometry data analysis.

Supplementary Figure S6. Correlation between IFNγ secretion from PBMCs stimulated with SARS-CoV-2 spike peptide pool and pseudovirus neutralization **(A)** or anti-S1 IgG levels **(B)** measured three months after administration of the second dose of the vaccine.

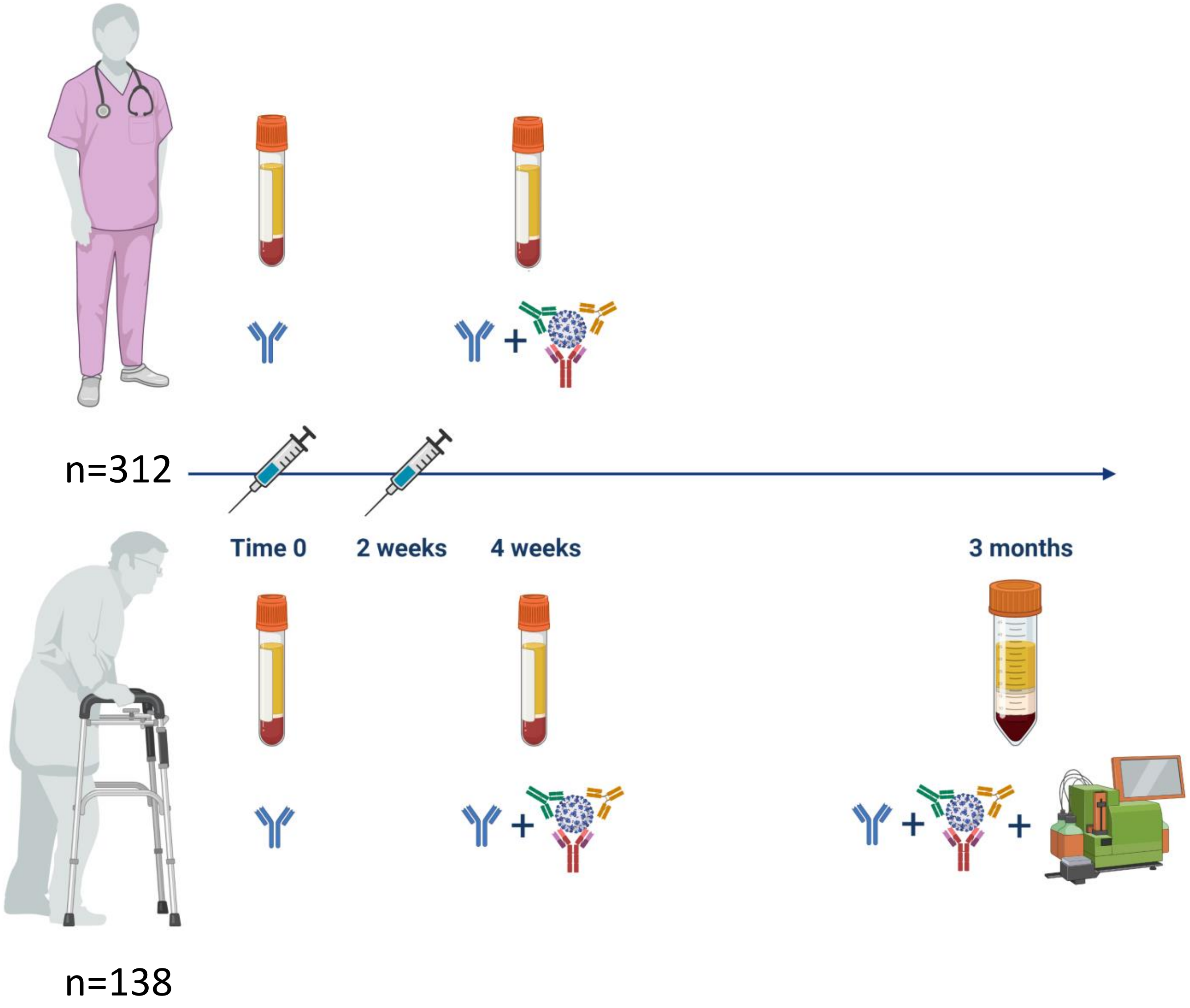

Figure S1.

A

	Number of participants	Median age (years)	anti-S IgG positive before vaccination	anti-NCP IgG positive before vaccination	double: anti-S/anti-N IgG positive before vaccination	positive in either of the assays before vaccination	anti-S IgG positive two weeks after second dose of vaccination
NHS	312	50,8	26 (8,3%)	26 (8,3%)	16 (5,1%)	36 (11,5%)	308 (98,7%)
NHR	138	88,9	28 (20,3%)	20 (14,5%)	19 (13,8%)	30 (21,7%)	122 (88,4%)

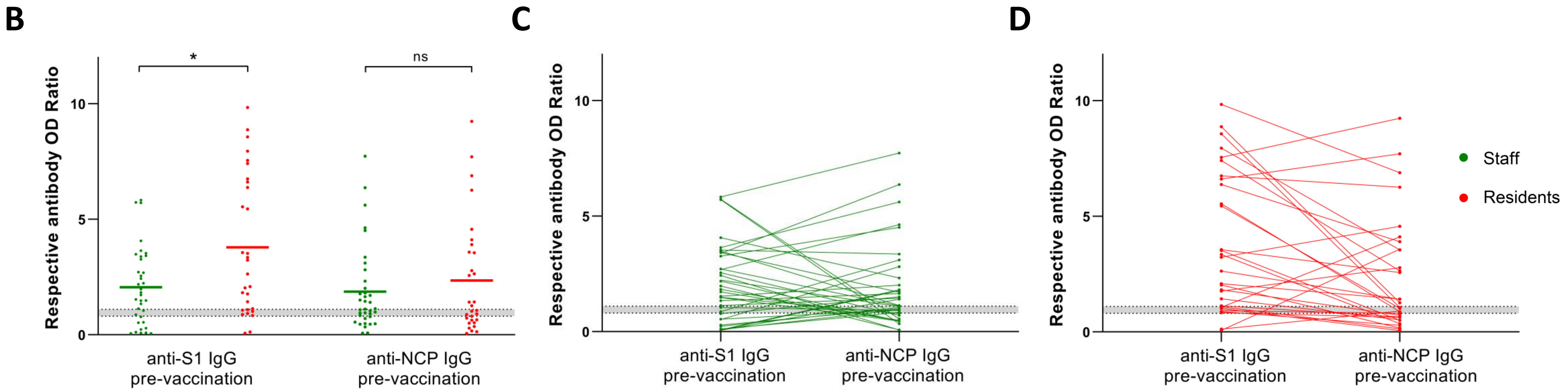

Figure S2.

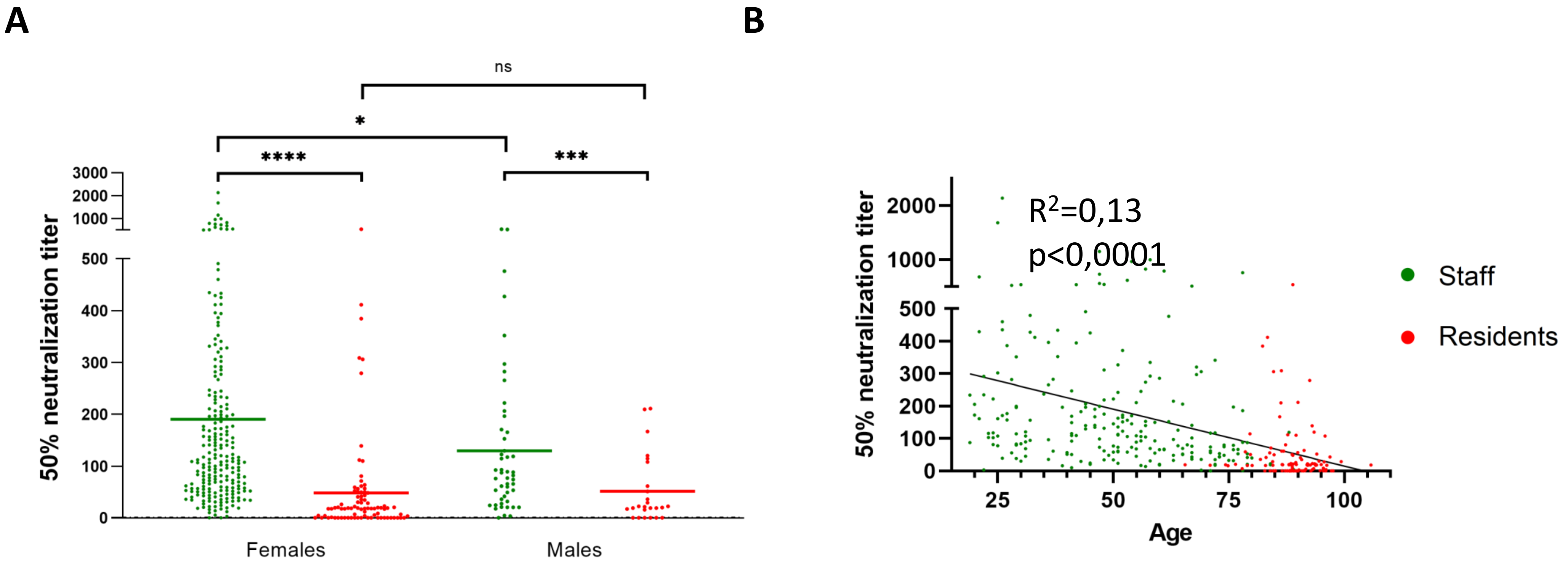
A
B
ns
*
****
***
3000
2000
1000
500
400
300
200
100
0
50% neutralization titer
Females
Males
R2=0,13
p<0,0001
2000
1000
500
400
300
200
100
0
25
50
75
100
Age
50% neutralization titer
Staff
Residents

Figure S3.

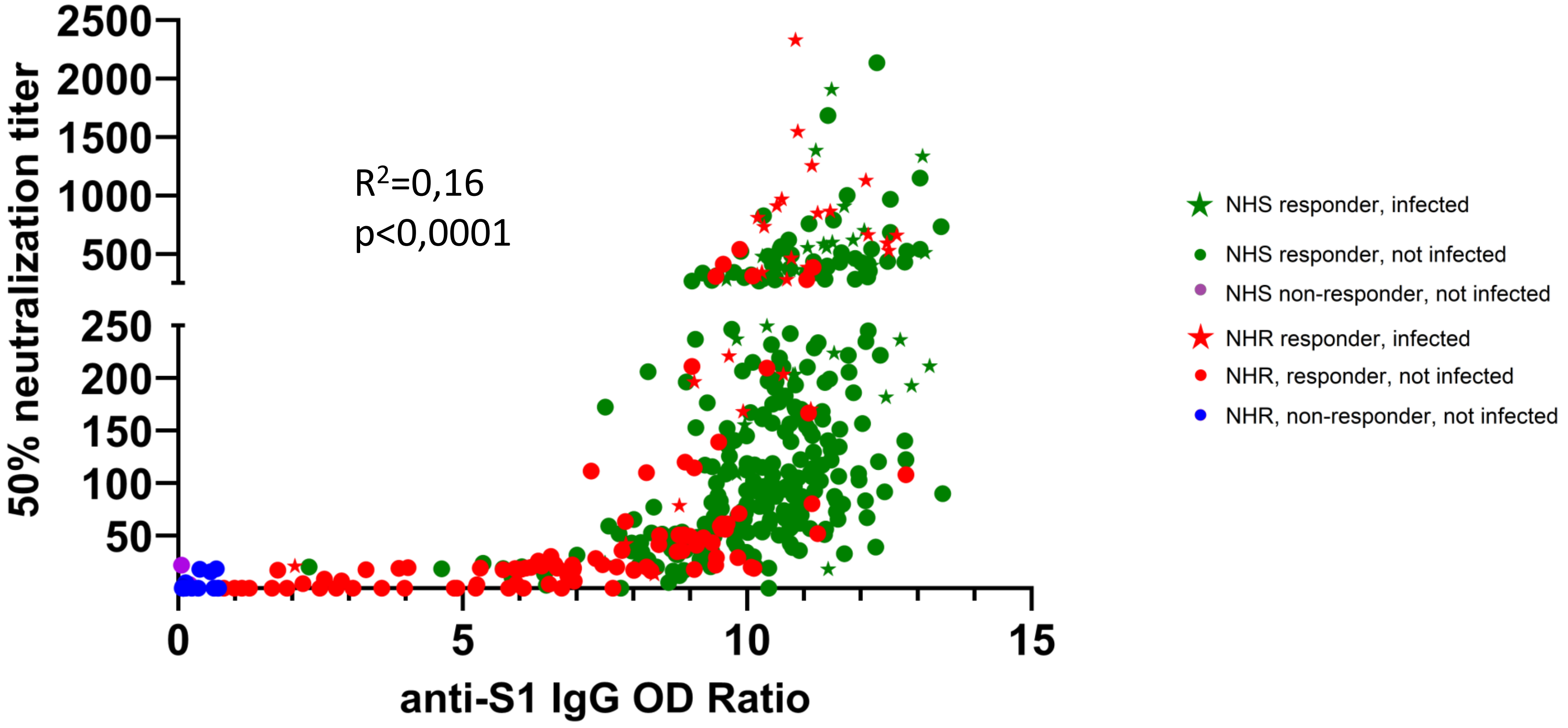

50% neutralization titer
2500
2000
1500
1000
500
250
200
150
100
50
0
5
10
15
anti-S1 IgG OD Ratio
R²=0,16
p<0,0001
NHS responder, infected
NHS responder, not infected
NHS non-responder, not infected
NHR responder, infected
NHR, responder, not infected
NHR, non-responder, not infected

Figure S4.

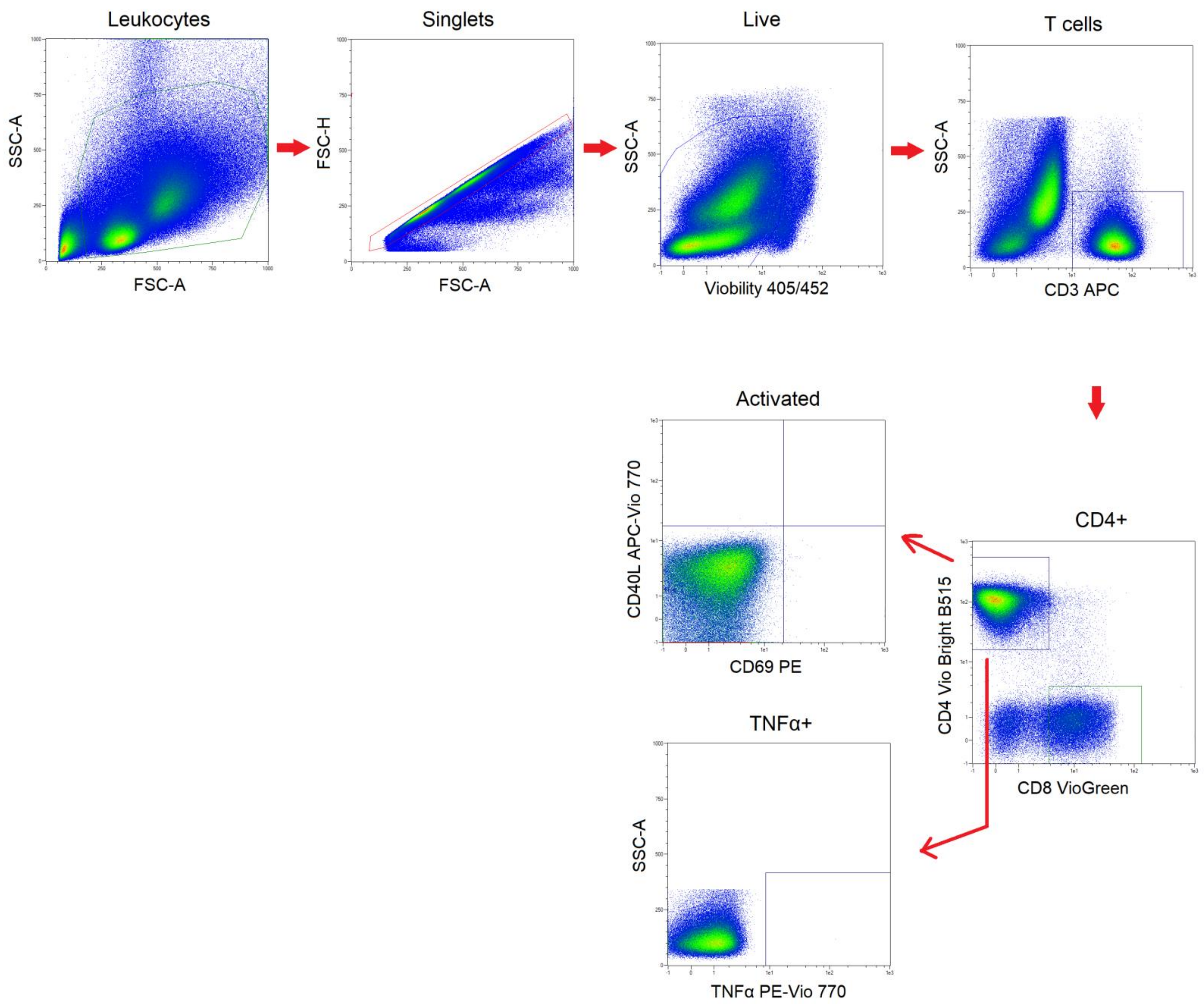
Leukocytes
SSC-A
FSC-A
Singlets
FSC-H
FSC-A
Live
SSC-A
Viobility 405/452
T cells
SSC-A
CD3 APC
Activated
CD40L APC-Vio 770
CD69 PE
CD4+
CD4 Vio Bright B515
CD8 VioGreen
TNFα+
SSC-A
TNFα PE-Vio 770

Figure S5.

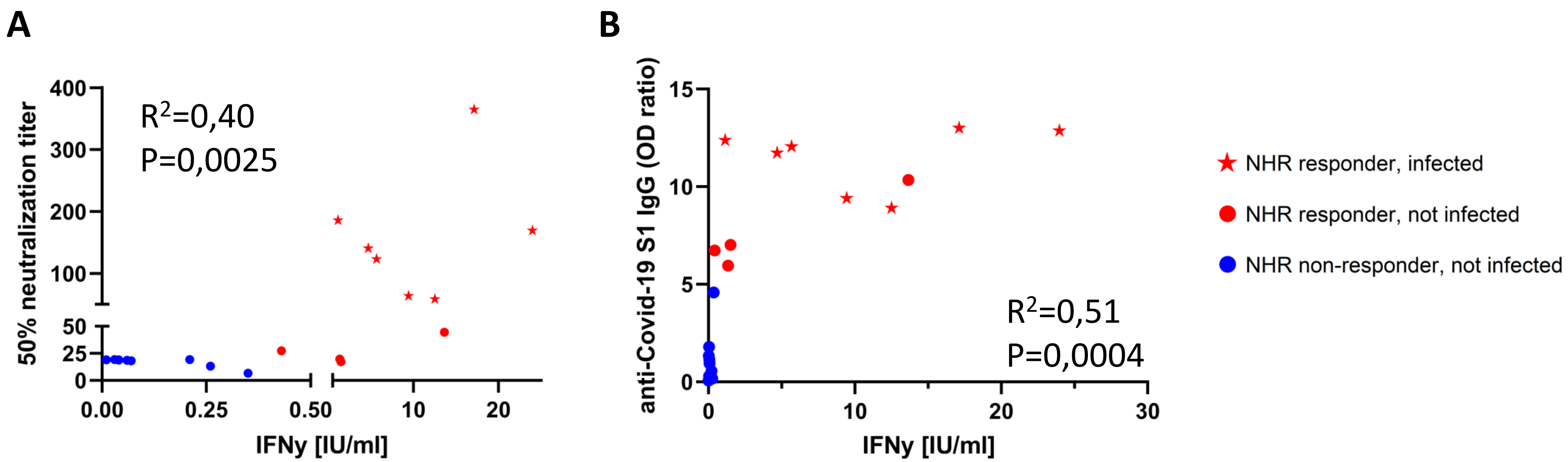
A
50% neutralization titer
400
300
200
100
50
25
0
R^2=0,40
P=0,0025
0.00
0.25
0.50
10
20
IFNy [IU/ml]
B
anti-Covid-19 S1 IgG (OD ratio)
15
10
5
0
R^2=0,51
P=0,0004
0
10
20
30
IFNy [IU/ml]
NHR responder, infected
NHR responder, not infected
NHR non-responder, not infected

Figure S6.
